# Construction of Chitosan-Zn-Based Electrochemical Biosensing Platform for Rapid and Accurate Assay of Actin

**DOI:** 10.3390/s18061865

**Published:** 2018-06-07

**Authors:** Chong Sun, Ye Zou, Daoying Wang, Zhiming Geng, Weimin Xu, Fang Liu, Jinxuan Cao

**Affiliations:** 1 Institute of Agricultural Products Processing, Jiangsu Academy of Agricultural Sciences, Nanjing 210014, China; sunchong@jaas.ac.cn (C.S.); zouye@jaas.ac.cn (Y.Z.); gengzhiming@jaas.ac.cn (Z.G.); xuweimin@jaas.ac.cn (W.X.); liufang@jaas.ac.cn (F.L.); 2 Jiangsu Key Laboratory for Food Quality and Safety-State Key Laboratory Cultivation Base of Ministry of Science and Technology, Nanjing 210014, China; 3 Key Laboratory of Animal Protein Food Processing Technology of Zhejiang Province, Ningbo University, Ningbo 315211, China

**Keywords:** chitosan-Zn nanoparticles, actin, electrochemical immunosensor, quality evaluation

## Abstract

This work reports a study on the development of a sensitive immunosensor for the assay of actin, which is fabricated using sensing material chitosan-Zn nanoparticles (NPs) and anti-actin modified on glassy carbon electrode respectively. The prepared materials were characterized using transmission electron microscope (TEM), fourier transform infrared spectra (FTIR), X-ray diffraction (XRD) spectra, and circular dichroism (CD) techniques. Meanwhile, the electrochemical properties were studied by linear sweep voltammetric (LSV), electrochemical impedance spectra (EIS), and differential pulse voltammetry (DPV). According to the experiments, under the optimum conditions, the linear fitting equation was I (μA) = −17.31 + 78.97c (R^2^ = 0.9948). The linear range was from 0.0001 to 0.1 mg/mL and the detection limit (LOD, S/N = 3) was 21.52 ng/mL. The interference studies were also performed for checking the sensors’ selectivity to actin. With better properties of the chitosan-Zn NPs, the modified electrode is considered as a better candidate than Western blot or immunohistochemical method for real-time usability. The detection limit reported is the lowest till date and this method provides a new approach for quality evaluation.

## 1. Introduction

Actin is the most abundant protein in most eukaryotic cells. It participates in many important physiological processes including muscle contraction, cell motility, cell division, and so on. In biomedicine fields, the actin cytoskeleton, as an extremely dynamic and highly ordered cell component, underpins abundant cellular processes and is vital for tumor proliferation and metastasis [1,2]. In the food industry, actin is predominantly linked to tenderness and texture of muscular tissues [3,4,5].

At present, the studies of actin are mainly in cytoskeleton structure and rearrangement [6,7,8,9]. However, little research has been focused on reliable and accurate detection of actin. Kim et al. designed a fluorescently labeled method for direct monitoring of actin utilizing a modified protein transduction tag from the HIV TAT sequence [10]. Shimozawa et al. used an actin filament labeled with either BODIPY TMR cadaverin-iodoacetamide to determine actin by fluorescence imaging [11]. Inamoto et al. used an immunohistochemical detection of actin by the mechanism of physical elimination [12]. However, some major drawbacks have been reported in these methods, for example, unfriendly environment, specific instruments, and complex operation. Western blot or immunohistochemical method has been the major protein detection tool. However, it is time-consuming because it usually requires 8–20 h to complete all the steps. The majority of steps are performed manually which makes them labor intensive, thus decreases reproducibility and quantification sensitivity [13]. Therefore, it is of great significance and urgency to develop an accurate, sensitive and facile method for actin determination. In contrast with these methods, electrochemical analysis was a superior method due to its low cost, fast response, good sensitivity and selectivity [14,15,16]. Thus, considerable efforts have been made to seek suitable materials for the design and construction of high-performance electrochemical sensor for actin determination.

Chitosan, as a natural biopolymer with abundant primary amino groups and hydroxyl groups, has a wide range of applications, such as drug delivery [17], supercapacitor [18], industrial additive [19,20] and environmental protection [21,22]. Due to its unique biological characteristics, for instance, nontoxicity and biocompatibility [23], chitosan has been gained comprehensive attention as a kind of modified electrode materials [24]. However, it does not conduct well. Thus, some metal oxide/metal nanoparticles [25,26] or two-dimensional laminar nanomaterials [27,28,29] were usually doped with chitosan to improve its high surface area, high intrinsic mobility and conductivity. With large surface area, good conductivity and biocompatibility, Zinc (Zn) was widely applied in solar cells, sensors and photocatalysis [30,31]. Zhao et al. presented self-assembly zinc oxide mesocrystal in chitosan for glucose biosensors [23]. Pandiselvi et al. synthesized inorganic-organic redox mediators of chitosan-ZnO/polyanilne nanocomposite for selective detection of dopamine [32]. Wang et al. prepared zinc complexes with a better antibacterial activity and excellent activity [33].

In this paper, chitosan-Zn nanoparticles (NPs) were designed and synthesized using sodium tripolyphosphate (STPP) as a surfactant, NaBH_4_ as a reducing agent and chitosan as a template. Furthermore, chitosan-Zn NPs were modified on the surface of glassy carbon electrode (GCE) and a novel immunosensor based on anti-actin was fabricated for monitoring of actin. More details of the preparation for modified electrode and the quantitative measurement of actin were presented.

## 2. Experiments Section

### 2.1. Reagents

Zinc nitrate hexahydrate (Zn(NO_3_)_2_·6H_2_O) was purchased from west gansu science Co. Ltd. (Shantou, China). Chitosan, acetic acid (HAc), sodium hydroxide (NaOH) and hemoglobin (Hb) were achieved from SCRC (China). NaBH_4_ was obtained from Sinopharm Chemical Reagent Co. Ltd. (Nanjing, China). Anti-actin, STPP, inosine monophosphate (IMP) and myoglobin (Mb) were purchased from Sigma-Aldrich (Darmstadt, Germany). The preparation of actin and heat shock protein 90 (HSP90) was presented in Part 2.7 and Part 2.8. Phosphate buffer solution (PBS) was prepared by mixing stock standard solution of NaCl, KCl, Na_2_HPO_4_ and KH_2_PO_4_. All other chemicals were of analytical reagent grade. Aqueous solutions were prepared with ultrapure water at room temperature.

### 2.2. Synthesis of Zn NPs

Zn NPs was synthetized with the co-precipitation method [34]. 20 mL Zn(NO_3_)_2_·6H_2_O (0.1 mol/L) was added in 10 mL NaOH (0.1 mol/L) under stirring for 2 h. Then, 5 mL NaBH_4_ (0.1 mol/L) was injected to the above solution for 4 h with continuous agitation at 60 °C. Finally, Zn NPs were obtained by washing the obtained precipitates with distilled water and being dried at 50 °C.

### 2.3. Synthesis of Chitosan-Zn NPs

The as-synthesized Zn NPs (0.03 g) were dispersed to 5 mL 0.1% chitosan solution (containing 1% HAc) for 30 min under agitation. 4 mL STPP (0.1%) was injected to the above solution and used as a surfactant, then the mixture was stirred for 5 h [35]. Finally, the obtained suspension was dialyzed (MW = 14,000) overnight, and then chitosan-Zn NPs was obtained. All the solutions were stored in a refrigerator at 4 °C.

### 2.4. Characterization

Transmission electron microscope (TEM) was characterized by a HITACHI H-7650 transmission electron microscopy (HITACHI, Tokyo, Japan) with an accelerating voltage of 80 kV. The samples were prepared by dropping the chitosan-Zn NPs solution onto a carbon-coated copper grid and dried at ambient temperature. Fourier transform infrared (FTIR) spectra (VARIAN Cary 5000, Agilent Technologies, Santa Clara, CA, USA) and X-ray diffraction (XRD) spectra (Smartlab, Tokyo, Japan) were measured to testify successful synthesis of chitosan-Zn NPs. The circular dichroism (CD) spectra (Applied photophysics, Leatherhead, UK) were collected from 180 to 280 nm to demonstrate the secondary structure of actin and the biocompatibility of chitosan-Zn NPs.

### 2.5. Preparation of Anti-Actin/Chitosan-Zn Modified GCE

GCE was polished with 0.3 and 0.05 µm aluminum oxide powder on a chamois leather, respectively. Afterwards, GCE was washed in anhydrous ethanol and ultrapure water under ultrasound for 30 s, and it was blown with nitrogen gas. 5 µL of chitosan-Zn NPs was dropped onto the surface of GCE and air-dried. Then, 5 µL of anti-actin (1 µmol/L) solution was immobilized on the surface of chitosan-Zn/GCE and dried in air. The anti-actin/chitosan-Zn/GCE was used in the following detection.

### 2.6. Electrochemical Detection

Electrochemical measurement was performed with a CHI 660D electrochemical workstation (Shanghai, China). All experiments were carried out with a conventional three-electrode system with GCE or modified GCE as the working electrode, a Pt wire electrode as the counter electrode and a saturated calomel electrode as the reference electrode.

The electrochemical behavior of GCE, chitosan-Zn/GCE, and anti-actin/chitosan-Zn/GCE was executed by linear sweep voltammetric (LSV) and electrochemical impedance spectra (EIS). LSV was tested in 0.1 mol/L PBS (PH = 7.0) containing 0.01 mg/mL actin with the scan rate of 100 mV/s. EIS was recorded in 5 mM [Fe(CN)_6_]^3−/4−^ (1:1) solution containing 0.1 M KCl with the initial potential of 0.22 V at the frequency range of 1 Hz–100 kHz with 5 mV AC amplitude. Differential pulse voltammetry (DPV) was carried out to detect the sensitivity of actin with the potential ranging from −0.1 to 0.6 V. The buffer solution was purged with high purity nitrogen prior to and blanked with nitrogen during the electrochemical experiments.

### 2.7. Preparation of HSP90

The process for preparing HSP90 was described as follows [36]. Biceps femoris muscles and livers were removed from goose meat and trimmed of all visible subcutaneous fat and connective tissues. 30 g of the sample was homogenized with 100 mL Tris-HCl buffer (100 mM, pH = 8.0) at 10,000 rpm using an Ultra Turrax (T25, IKA, Breisgau, Germany). The homogenate was then centrifuged for 20 min at 12,000 × *g* (Allegra 64R, Beckman, CA, USA), supernatant was collected and filtered through four-layer gauze. Afterwards, the filtrate was precipitated by ammonium sulphate at a saturation of 70% at 4 °C for 12 h under gentle stirring, and centrifuged for 20 min at 12,000 × *g*, the precipitate was dissolved in a minimal volume of Tris-HCl buffer (100 mM, pH = 8.0). The HSP90 solution was dialyzed for 24 h in Tris-HCl buffer (100 mM, pH = 8.0), then lyophilized and stored at −20 °C.

### 2.8. Preparation and Determination of Actin

The extraction of actin was performed according to our previous method with slight modifications [37]. 2 g of goose breast meat was mixed with 25 mL of Weber-Edsall solution (0.6 M KCl, 0.01 M Na_2_CO_3_, 0.04 M NaHCO_3_, pH = 7.2) and homogenized at 12,000 *g* for 30 s at intervals of 10 s in ice-water bath. The resulting homogenate was transferred to a test tube and was shaken for 24 h at 4 °C. The total actin solution was obtained from the homogenate by removing the insoluble substances with two layers of nylon mesh filter. Then, 10 mL of the actin solution was mixed with 20 mL of distilled water to lower the KCl concentration to 0.2 M, and the solution was centrifuged at 15,000 *g* for 20 min at 4 °C. The supernatant was the actin solution.

Sodium dodecyl sulfate-polyacrylamide gel electrophoresis (SDS-PAGE) was conducted according to our previous method described in the literature [37]. The actin solutions collected in the previous step were added to an equivalent volume of reduced sample buffer (62.5 mM Tris-HCl (pH = 6.8), 10% glycerol, 2% SDS, 5% 2-mercaptoethanol, 0.02% bromophenol blue), then it was heated in boiling water for 5 min. The solutions mixed with the reduced sample buffer were placed onto discontinuous 8% polyacrylamide gel. 15 µL of the sample were separated using a vertical gel electrophoresis device (PowerPac^TM^, Bio-Rad, Hercules, Singapore) at 120 V and 20 mA.

The control experiment was conducted using Bradford method to determine actin concentration, which was monitored spectrophotometrically at 595 nm with bovine serum albumin as the standard.

## 3. Results and Discussion

### 3.1. Characterization

**Figure 1:**
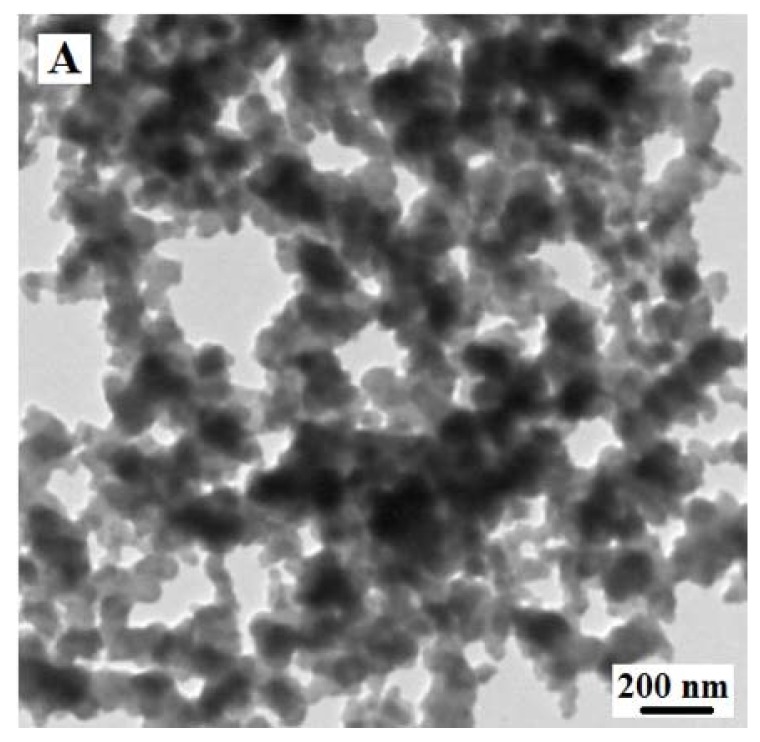

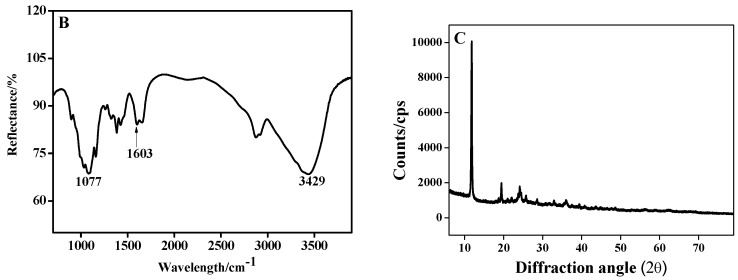
(**A**) Transmission electron microscope (TEM) image of chitosan-Zn nanoparticles (NPs); (**B**) fourier transform infrared spectra (FTIR) spectra of chitosan-Zn NPs; (**C**) X-ray diffraction (XRD) spectra of chitosan-Zn NPs.

TEM was performed to characterize the structure and morphology of chitosan-Zn NPs. As displayed in Figure 1A, it was demonstrated that chitosan formed a template and Zn was firmly and uniformly attached to the surface of chitosan.

FTIR spectra and XRD were tested to prove successful synthesis of chitosan-Zn NPs. Figure 1B showed the FTIR spectra of chitosan-Zn NPs. The strong peak at 3429 cm^−1^ was attributed to N-H stretching vibration of amide groups and O-H stretching vibration. The weak peak at 1603 cm^−1^ was assigned to N-H flexural vibration of amide groups. The peak at 1077 cm^−1^ corresponded to the second hydroxy groups [33,34]. The results indicated that CS had been trapped into chitosan-Zn NPs successfully.

Figure 1C illustrated the XRD of the chitosan-Zn NPs. The XRD pattern exhibited diffraction peaks at 10.2° and 19.8° were due to the presence of polyacrylamide in the chitosan backbone. Three different peaks were at 2θ = 15.89°, 30.1° and 32.87°, which clearly indexed as the cubic Zn phase [34]. These data are in consistent with TEM and FTIR spectra. Therefore, chitosan-Zn NPs were successfully prepared.

### 3.2. SDS-PAGE of the Extracted Actin

**Figure A1:**
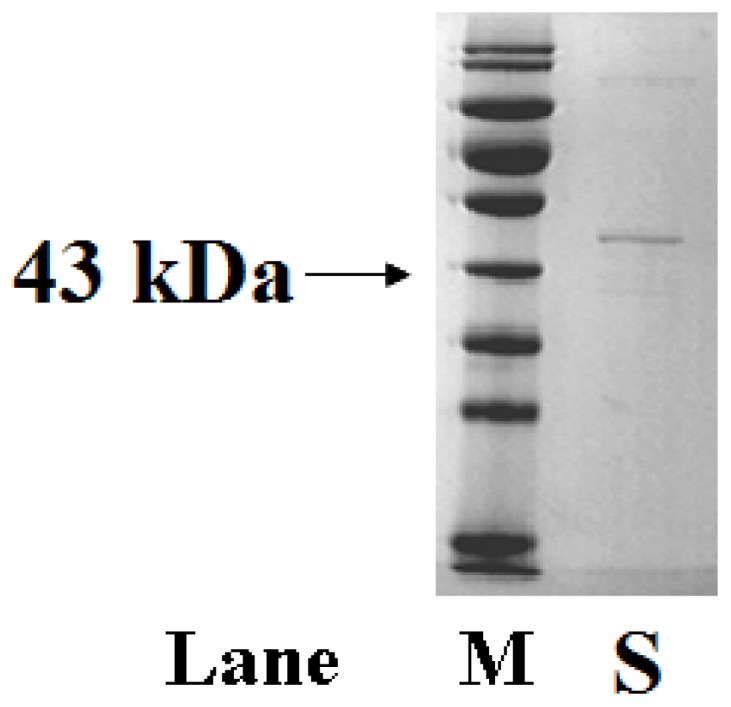
Sodium dodecyl sulfate-polyacrylamide gel electrophoresis (SDS-PAGE) images of the extracted actin. (Lane M): Protein marker, (Lane S): actin.

The SDS-PAGE profiles of the samples were shown in Figure A1. The band of the exacted actin solution (Figure A1, lane S) was clear and aligned to the corresponding protein marker of 43 kDa (Figure A1, lane M). This suggested that actin was exacted from goose breast meat successfully.

### 3.3. Biocompatibility

**Figure 2:**
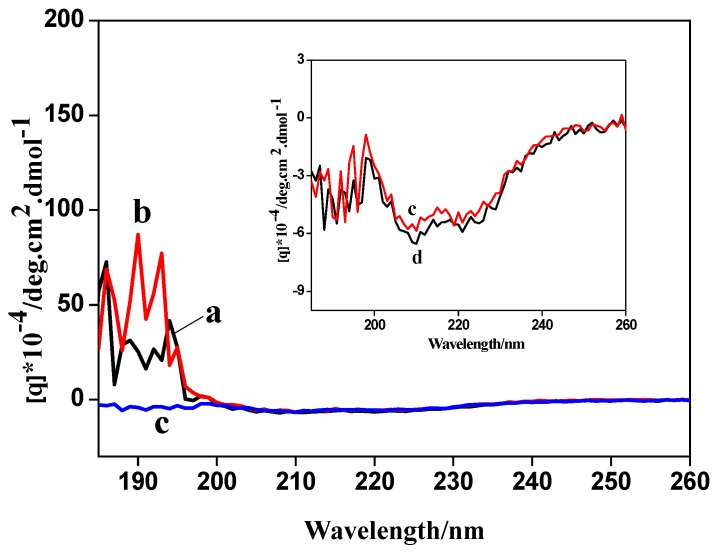
Circular dichroism (CD) spectra of (a) actin, (b) anti-actin, (c) actin/anti-actin and (d) actin/anti-actin/chitosan-Zn.

CD spectra were used to investigate the secondary structure of protein. CD spectra of actin (curve a), anti-actin (curve b), actin/anti-actin (curve c) and actin/anti-actin/chitosan-Zn (curve d) were shown in Figure 2. CD spectra of actin/anti-actin had an obvious difference in peak shape and the peak height with CD spectra of actin and anti-actin, indicating that actin and anti-actin have bound to change the secondary structure of actin. In contrast with actin/anti-actin, actin/anti-actin/chitosan-Zn had no obvious changes in the peak shape and the peak height, demonstrating that chitosan-Zn NPs had good biocompatibility.

### 3.4. Optimization of Experimental Parameters

**Figure 3:**
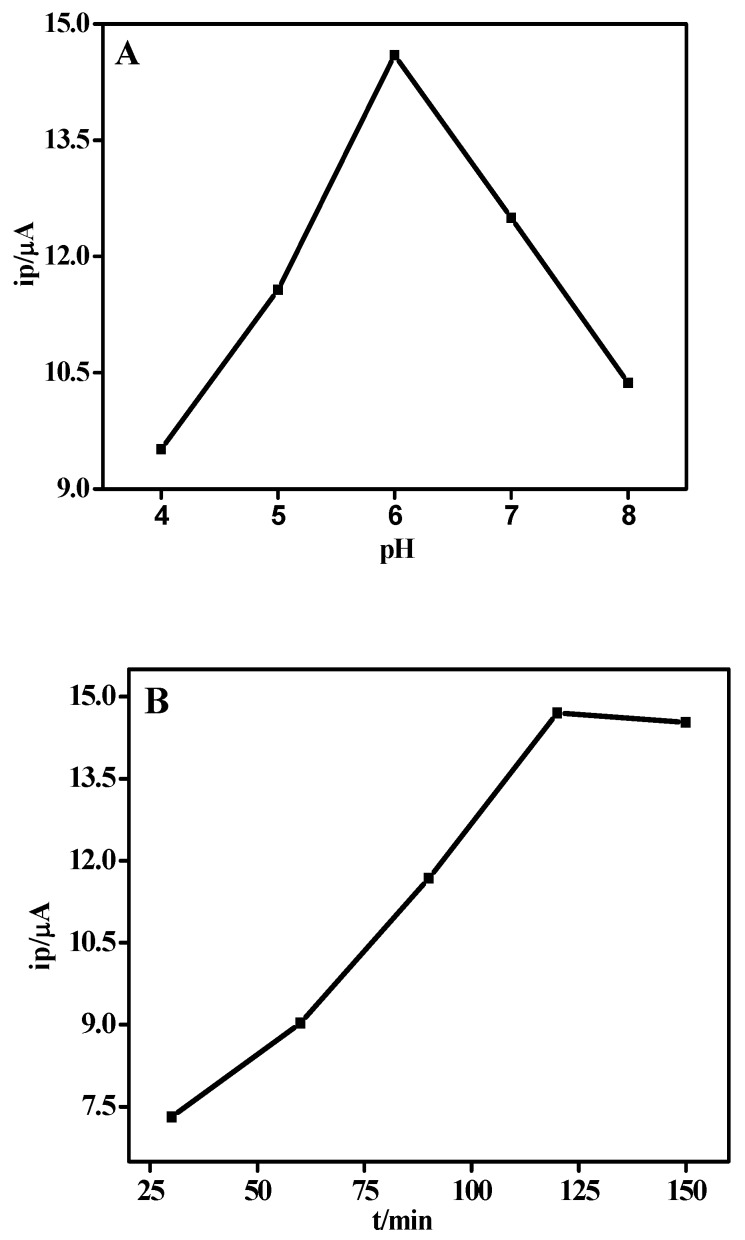

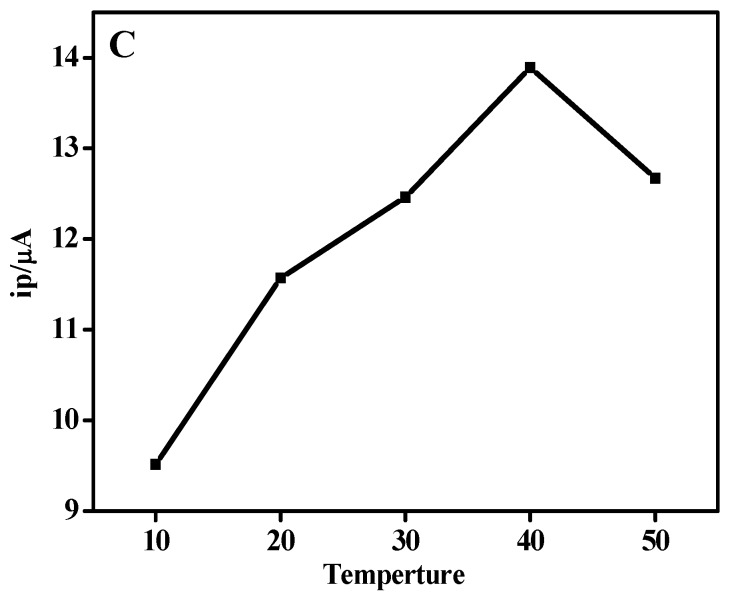
The plots for the relationship of (**A**) the pH value, (**B**) binding time and (**C**) temperature with electrochemical signal in 0.1 M PBS (pH = 7.0).

The experimental parameters, including pH values, binding time and temperature, were investigated by DPV method, and the results were shown in Figure 3. The prepared actin/anti-actin/chitosan-Zn/GCE immunosensor was detected for the dependence of peak current on the pH (5, 6, 7, 8 PBS) and pH = 4.0 (citrate buffer). From the plot for the relationship between the pH values and the peak currents in Figure 3A, it was observed that the peak current increased with pH value ranging from 4 to 6, and then it decreased when the pH value is over 6. The modified electrode had a better sensitivity at 6.0 of the pH value. The binding time and temperature exerted a great influence on the hatchability of actin on the surface of modified electrode. Actin was incubated with anti-actin/chitosan-Zn/GCE at different time (30, 60, 90, 120, 150 min). Then, the modified electrodes were determined for the dependence of peak current on the adsorption time. Figure 3B showed the plot for the relationship between the binding time and the peak currents. With the binding time elongating, the peak current increased and remained stable after 120 min, indicating that 120 min was the optimal time. The thermostatic water bath was used to control temperature. Actin was incubated with anti-actin/chitosan-Zn/GCE at different temperature (10, 20, 30, 40, 50 °C). Then, the prepared actin/anti-actin/chitosan-Zn/GCE immunosensor was detected for the dependence of peak current on the adsorption temperature. Figure 3C presented the plot for the relationship between temperature and the peak currents. The peak current value first increased and achieved the maximum with temperature at 40 °C, and then it began to decrease. Therefore, 40 °C was selected in the following detection.

### 3.5. Electrochemical Behavior of Modified Electrode

**Figure 4:**
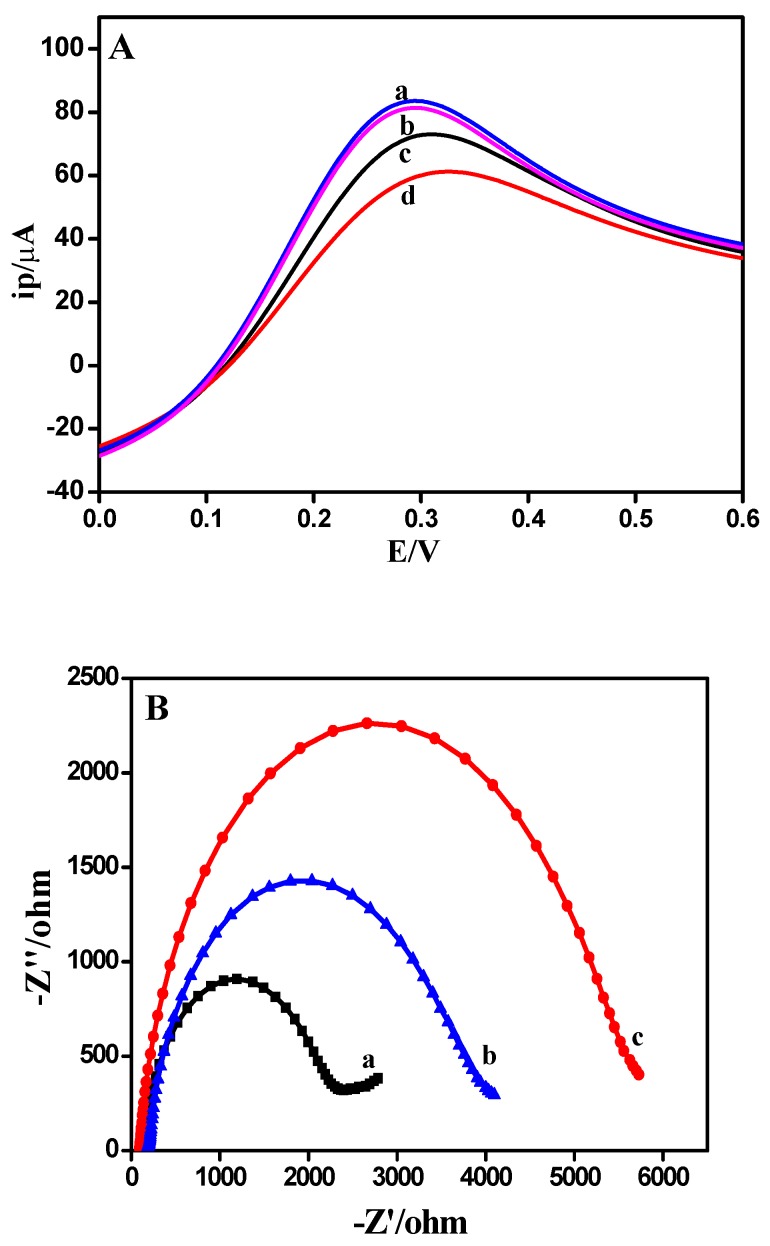
(**A**) Linear sweep voltammetrics (LSVs) of (a) chitosan-Zn/GCE, (b) actin/chitosan-Zn/GCE, (c) anti-actin/chitosan-Zn/GCE and (d) actin/anti-actin/chitosan-Zn/GCE in 0.1 M PBS (pH = 7.0). (**B**) Electrochemical impedance spectra (EISes) of (a) chitosan-Zn/GCE, (b) anti-actin/chitosan-Zn/GCE and (c) actin/anti-actin/chitosan-Zn/GCE in 0.1 M PBS (pH = 7.0) containing 5 mM [Fe(CN)_6_]^3−/4−^ (1:1) solution and 0.1 M KCl. Scan rate: 100 mV/s.

LSV was measured to study the electrochemical characteristics of the fabricated immunosensor. LSVs of chitosan-Zn/GCE (curve a), actin/chitosan-Zn/GCE (curve b), anti-actin/chitosan-Zn/GCE (curve c) and actin/anti-actin/chitosan-Zn/GCE (curve d) were investigated and the results were represented in Figure 4A. In the presence of actin, anti-actin/chitosan-Zn/GCE bound to actin, a complex of anti-actin-actin was formed and such a complex increased the steric hindrance that greatly attenuating the electron transfer between soluble redox couple. When actin was dropped onto the surface of chitosan-Zn/GCE, the peak current decreased slightly instead. The experiment indicated that actin could not attach directly to the chitosan-Zn/GCE. Compared with chitosan-Zn/GCE, the peak current of anti-actin/chitosan-Zn/GCE decreased remarkably, indicating that anti-actin was successfully immobilized on the modified electrode and anti-actin had a poor conductivity. After the incubation of actin, the peak current further decreased, because the introduction of actin formed a steric hindrance process to hinder electron transfer.

EIS was conducted to investigate the properties of different electrode materials and the diameter of the semicircle in EIS was closely related to the resistant charge transfer [38]. EISes of chitosan-Zn/GCE (curve a), anti-actin/chitosan-Zn/GCE (curve b) and actin/anti-actin/chitosan-Zn/GCE (curve c) were presented in Figure 4B. Anti-actin/chitosan-Zn/GCE expressed a larger diameter than chitosan-Zn/GCE, owing to the interface electron-transfer impedance of anti-actin. After being reacted with actin, the diameter of actin/anti-actin/chitosan-Zn/GCE became larger, due to the presence of actin. The results of CV and EIS demonstrated that the modified electrode of actin/anti-actin/chitosan-Zn/GCE was successfully prepared.

### 3.6. Actin Determination

**Figure 5:**
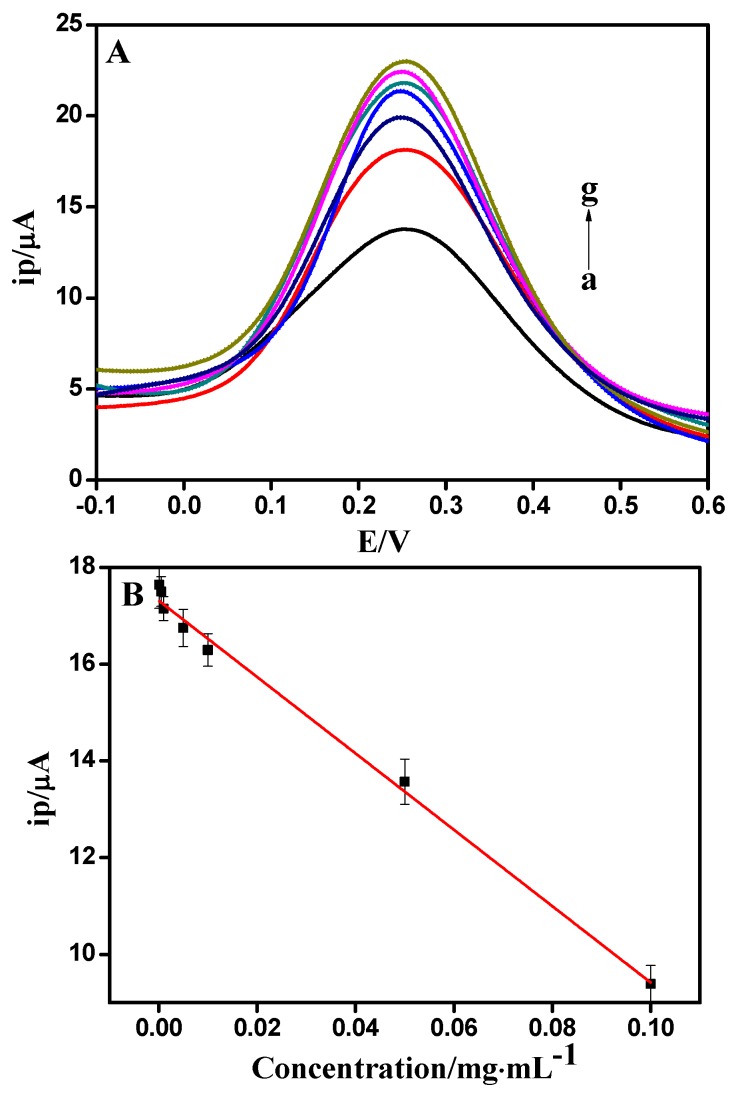
(**A**) Differential pulse voltammetry (DPV) obtained at anti-actin/chitosan-Zn/GCE in 0.1 M PBS (pH = 7.0) with the concentration of actin (from a to g) 0.1, 0.05, 0.01, 0.005, 0.001, 0.0005, 0.0001 mg/mL. (**B**) Calibration curve for anti-actin/chitosan-Zn/GCE.

Due to a low detection limit and highly sensitive electrochemical method, DPV was carried out for the quantitative determination of actin. Figure 5A showed the DPVs of actin/anti-actin/chitosan-Zn/GCE in 0.1 mol/L PBS (pH = 7.0) and various concentration of actin (0.1, 0.05, 0.01, 0.005, 0.001, 0.0005, 0.0001 mg/mL). It is showed that the peak current gradually decreased with increasing concentrations of actin. Note that the redox potential shifted to be negative with increasing actin concentrations. A possible explanation is that there are changes in the charged surface of the immunosensor and its electronegativity may decrease upon interaction with actin. Thus, its repellence to redox probe is reduced, leading to a negative shift of the redox potential. It is obvious that the peak currents were linear proportional to the concentration of actin in the range from 0.0001 to 0.1 mg/mL. As shown in Figure 5B, the linear regression equation was I (μA) = −17.3137 + 78.9691c (R^2^ = 0.9948), with the detection limit of 21.52 ng/mL.

### 3.7. Interference

**Figure 6:**
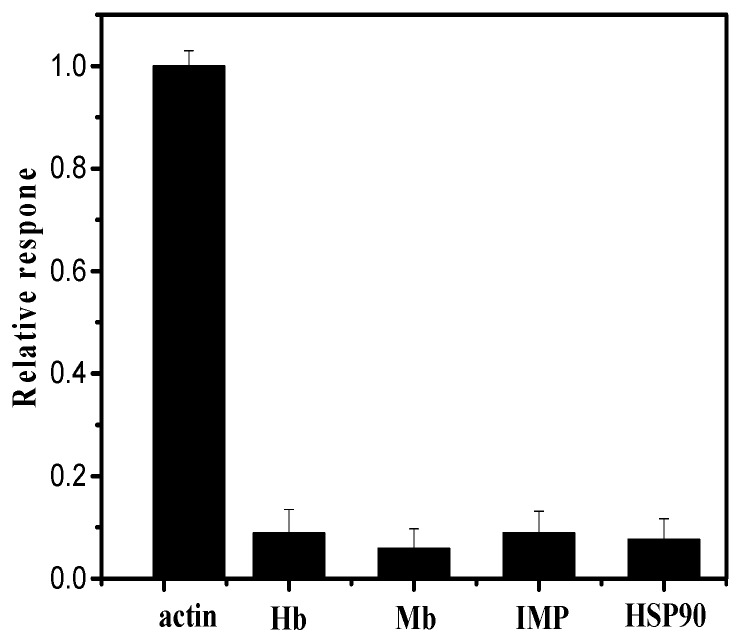
Detecting interference in solution containing Hb, Mb, IMP and HSP90 respectively. It is an average value of the response of three measurements for each sample by defining the signal of actin as 100%.

Hb, Mb, IMP, and HSP90 were considered as the interfering substances, because they were closely relevant to meat quality evaluation. Figure 6 represented the results of the interference of Hb, Mb, IMP and HSP90. It was found that all the test interferences showed no obvious interference to the detection of actin, illustrating that anti-actin/chitosan-Zn/GCE possessed an excellent anti-interference behavior towards actin.

### 3.8. Sensing Applications for Real Samples

The practical application performance of the anti-actin/chitosan-Zn/GCE sensing platform was confirmed by detection of actin in meat. Bradford method was used as a reference method. According to the obtained calibration curve, the detection results of four different batches of samples were listed in Table 1, which were in good agreement with the results provided by Bradford method. Moreover, the usefulness of the immunosensor was determined by recovery tests. As indicated in Table 2, the fabricated immunosensor had a good recovery. Thus, the immunosensor can be applied in practical samples satisfactorily and efficiently.

## 4. Conclusions

In summary, a rapid, accurate and convenient approach was described for actin analysis based on the construction of chitosan-Zn NPs and anti-actin based electrochemical biosensing platform. Taking advantage of the large surface to volume ratio of chitosan-Zn NPs, a mass of anti-actin was conducive to being bound on the modified electrode, and the electrochemical signal was clearly acquired at the immunosensor without the need for designing complicated process, which significantly accelerated the analysis speed. Compared with other methods, the immunosensor demonstrated wide linearity, high sensitivity, and good selectivity. The present work implemented the assay of actin in real samples with acceptable results, which illuminated the practical application fields of the sensing platform.

## Figures and Tables

**Table 1:** Comparison of Bradford method and the immunosensor obtained in practical samples.

Samples	Muscle-1	Muscle-2	Muscle-3	Muscle-4
Bradford method (mg/g)	33.5 ± 3.82	37.4 ± 4.11	29.4 ± 3.45	30.3 ± 4.15
Immunosensor (mg/g)	34.6 ± 2.15	38.5 ± 2.56	30.1 ± 2.14	31.0 ± 2.72

**Table 2:** Determination of actin in goose, duck and chicken breast meat (*n* = 6).

Samples	Found before Adding (mg/g)	Added (mg/g)	Found after Adding (mg/g)	Recovery (%)	RSD (*n* = 6)
Goose breast meat	32.3	10.0	41.7	98.6	2.03
Duck breast meat	33.5	10.0	42.4	97.4	1.96
Chicken breast meat	30.1	10.0	39.2	97.8	2.23
